# Shear Behavior and Predictive Model of Desert Sand Concrete Beams Subjected to Freeze–Thaw Cycles

**DOI:** 10.3390/ma19132721

**Published:** 2026-06-25

**Authors:** Chao Huang, Meng Wu, Zhiqiang Li, Yingsheng Dang, Jian Li

**Affiliations:** 1 College of Water Conservancy & Architectural Engineering, Shihezi University, Shihezi 832003, China; 19990213986@163.com (C.H.); lijian8.14@163.com (J.L.); 2 College of Safety Science and Engineering, Xinjiang Institute of Engineering, Urumqi 830023, China

**Keywords:** desert sand concrete, freeze-thaw cycles, diagonal cracks, shear behavior, shear capacity

## Abstract

To explore the shear behavior and evolutionary pattern of desert sand concrete beams (DSCBs) subjected to freeze–thaw cycles, 16 DSCBs were subjected to rapid freeze–thaw cycling and shear tests, with desert sand replacement ratios (0%, 20%, 40%, and 60%) and numbers of freeze–thaw cycles (0, 25, 50, and 75) considered as the main variables. The failure mode, diagonal crack development, diagonal cracking load, shear capacity, and load–stirrup strain curves of DSCBs were tested and analyzed. The results indicate that all specimens exhibited typical shear-compression failure. The diagonal crack development pattern of DSCBs was similar to that of ordinary concrete beams, whereas freeze–thaw cycles accelerated the initiation and propagation of cracks. Freeze–thaw cycling significantly reduced both the diagonal cracking load and shear capacity. After being exposed to 75 cycles of freezing and thawing, the ultimate shear capacity of test pieces with desert sand replacement proportions of 0%, 20%, 40%, and 60% decreased by 15.6%, 12.9%, 13.9%, and 13.8%, respectively, while the corresponding stirrup strains increased by 47.2%, 34.1%, 37.1%, and 53.7%, respectively. An appropriate desert sand replacement ratio can improve the shear performance of concrete beams. Among all specimens, the beam with a 20% replacement ratio exhibited the best overall mechanical performance, achieving a maximum increase of 6.0% in shear capacity and a maximum reduction of 26.8% in stirrup strain compared with conventional concrete beams. Finally, by introducing modification coefficients related to the desert sand replacement ratio as well as the freeze–thaw cycling times, predictive equations for the diagonal cracking load and shear capacity of DSCBs under freeze–thaw conditions were established. The numerical predictions achieve a high consistency with measured data.

## 1. Introduction

In contemporary civil engineering, concrete serves as one of the predominant materials for infrastructure development, and its production and application heavily depend on natural sand and gravel aggregates. However, in the context of booming global urbanization and growing-scale engineering development, natural river sand resources are becoming increasingly scarce, resulting in more pronounced ecological and resource supply–demand conflicts [1]. The development and utilization of alternative fine aggregates have become an important approach to achieve greener and more sustainable concrete materials [2,3,4,5,6]. Desert sand is abundant and widely distributed; statistics show that the global desert area exceeds 36 million km^2^, with a substantial reserve located in northwest China alone. Therefore, incorporating desert sand into concrete production can mitigate the lack of natural river sand resources, facilitate the efficient reuse of desert materials, and cut down overall engineering expenditures [7,8,9,10,11].

Due to prolonged wind erosion, desert sand particles are rounded or elliptical, have relatively smooth surfaces, generally exhibit lower fineness modulus than natural river sand, and contain a higher proportion of fine particles [12]. In recent years, extensive research has been conducted internationally and domestically on the workability, mechanical properties, microstructural mechanisms, and durability of desert sand concrete (DSC). Hamada et al. [13] reported that the compressive strength of concrete reached its maximum at a 50% desert sand replacement ratio, but decreased when the replacement exceeded 25%. Xu et al. [14] developed a stochastic damage evolution model for uniaxial compression of full desert sand high-performance concrete based on the Weibull distribution and effectively predicted damage parameters using mix proportions. Zhang et al. [15] applied desert sand in ultra-high-performance alkali-activated concrete; at a 10% volumetric replacement, compressive strength increased by approximately 20% compared to natural silica sand, whereas excessive replacement reduced interfacial transition zone micro-mechanical performance due to increased fine particles. Shen et al. [16] investigated the high-temperature performance of DSC, showing that a 20–40% replacement ratio led to greater strength improvement than ordinary concrete. Wang et al. [17] prepared engineered cementitious composites using desert sand and local PVA fibers; at 100% fine aggregate replacement, tensile ductility was significantly improved. Liu et al. [18] studied the erosion characteristics of concrete under wind-sand load, proposed models for erosion rate and dynamic modulus degradation, and developed a digital image-based method for surface erosion assessment.

In cold regions, concrete structures are exposed to long-term freeze–thaw cycles during service. Free water within the pores expands by approximately 9% upon freezing, and the resulting frost heave and pore pressure induce microcrack initiation and propagation. With increasing cycles, concrete undergoes mass loss and gradual reduction in dynamic modulus and strength, while the interfacial transition zone, as a mechanically weak region, becomes the preferential site for crack development and damage accumulation [19]. Previous studies have indicated that freeze–thaw degradation in concrete is governed by intrinsic factors, including pore structure, moisture content, and aggregate type [20]. Arasteh-Khoshbin et al. [21] through freeze–thaw damage analysis and crack simulation, found that the crack development process under freeze–thaw environments exerts a significant influence on the long-term durability of concrete. The initiation and propagation of cracks promote the continuous accumulation of internal damage, ultimately leading to pronounced deterioration of material properties. This study revealed the development law of freeze–thaw damage from the perspective of crack evolution, thereby offering new research insights into the performance degradation of concrete structures subjected to freeze–thaw cycles. Arasteh-Khoshbin et al. [22] also investigated the effects of supplementary cementitious materials, including nano-SiO_2_, nano-Al_2_O_3_, and rice husk ash, on the mechanical properties and durability of concrete. The results indicated that nanomaterials and mineral admixtures can effectively reduce the permeability and chloride ion diffusivity of concrete, while simultaneously improving its compressive strength and durability. The study attributed these enhancements primarily to the micro-filling effect, refinement of the pore structure, and increased compactness of the cementitious matrix, which are considered critical factors in improving concrete durability. Experimental research has confirmed that freeze–thaw cycles accelerate the propagation of pre-existing cracks, leading to exponential decay of structural mechanical properties and altered microcrack patterns. Techniques such as air-entrainment modification, fiber reinforcement, and surface treatment can effectively enhance freeze–thaw resistance, and machine learning algorithms have been employed to quantify microscale damage parameters [23,24,25,26].

Most existing investigations into the freeze–thaw resistance of DSC pay attention to material-scale analysis. Gong et al. [27] found through freeze–thaw tests that when desert sand is blended with manufactured sand, a 40% desert sand content provides optimal frost resistance. Pan et al. [28] incorporated steel fibers to improve the cracking resistance of DSC and found that a 1.0% volumetric addition of steel fibers significantly enhanced post-freeze–thaw fracture behavior and delayed degradation. Li et al. [29] investigated the durability deterioration mechanisms of aeolian sand concrete under combined carbonation and freeze–thaw effects, observing that higher aeolian sand content improved frost resistance. Dong et al. [30] applied fractal theory to investigate the capillary water absorption of DSC under sulfate freeze–thaw coupling, suggesting that appropriately increasing the proportion of pores smaller than 50 nm enhances frost durability, and higher aeolian sand content reduces initial water absorption. Luo et al. [31] studied the freeze–thaw damage mechanism of aeolian sand concrete via macro and microscopic experiments, finding that a 30% aeolian sand replacement ratio resulted in optimal frost resistance.

Beams are among the most fundamental and important components in reinforced concrete structures, and their shear performance is directly related to structural safety reserves and failure modes. Compared with flexural failure, shear failure is typically brittle, sudden, and lacks early warning, making shear performance a key focus in reinforced concrete research. A large number of experimental investigations have explored the shear mechanical properties of concrete structural members in recent years. Li et al. [32] conducted tests on the shear characteristics of DSCBs and analyzed the influences of shear span-to-depth ratio, stirrup ratio and aeolian sand replacement rate, before developing a quantitative formula for shear strength calculation. Lu et al. [33] explored the residual shear bearing performance of corroded reinforced concrete beams and formulated an updated shear capacity model through the introduction of reduction coefficients. Kueres et al. [34] focused on the shear resistance of prestressed concrete beams strengthened with FRP reinforcements and established a simplified design approach for evaluating the shear performance of such structural components. Kim et al. [35] assessed the shear behavior of broad concrete beams with GFRP bars adopted as shear reinforcements, and refined the existing shear strength prediction formula by accounting for the influence of transverse reinforcement spacing. Wardeh et al. [36] tested the shear response of non-stirrup concrete beams fabricated with full recycled coarse aggregates. Their test outcomes demonstrated that recycled aggregate concrete possesses inferior splitting tensile strength, fracture energy and shear resistance. Mohammed Ali et al. [37] explored the impacts of PET fiber dosage on the shear resistance and deformation performance of reinforced concrete beams, and derived corresponding computational formulas for shear strength prediction.

Although existing studies have extensively investigated the mechanical properties, durability, and freeze–thaw damage mechanisms of desert sand concrete (DSC), the current findings are predominantly focused on material-level performance evaluations. Even those studies conducted at the structural level have been carried out under ordinary environmental conditions. Research on the structural behavior of desert sand concrete beams (DSCBs) under freeze–thaw environments, particularly concerning diagonal-section cracking behavior, crack development patterns, and shear bearing capacity degradation mechanisms, remains relatively insufficient. Furthermore, the existing calculation methods for the shear bearing capacity of concrete beams are primarily established based on conventional concrete members, and their design parameters have not yet accounted for the combined effects of desert sand replacement ratio and freeze–thaw damage on the tensile properties of concrete, crack propagation characteristics, and the mechanical mechanism of diagonal sections. Consequently, whether the current design methods are applicable to DSCB subjected to freeze–thaw environments still lacks systematic experimental verification. In view of the above research status, this study conducts shear performance tests on DSCB after freeze–thaw cycles, with desert sand replacement ratio and the number of freeze–thaw cycles as variables. The shear performance is emphatically analyzed, and the mechanical characteristics of the beams are revealed from the perspectives of diagonal crack features, diagonal-section cracking load, stirrup strain, and shear bearing capacity. Based on the current Chinese Code for Design of Concrete Structures [38], calculation formulas for the diagonal-section cracking load and shear bearing capacity of DSCB under freeze–thaw environments are established, with the aim of providing a theoretical reference for the promotion of DSC in engineering practice.

## 2. Experimental Program and Methodology

### 2.1. Raw Materials and Mix Proportion

**Figure 1:**
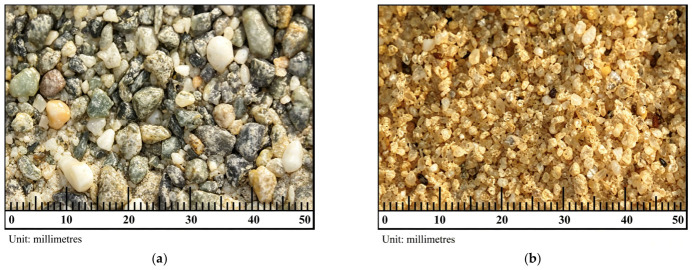
Apparent morphology of fine aggregate: (**a**) river sand; (**b**) desert sand (reproduced from [39]).

**Figure 2:**
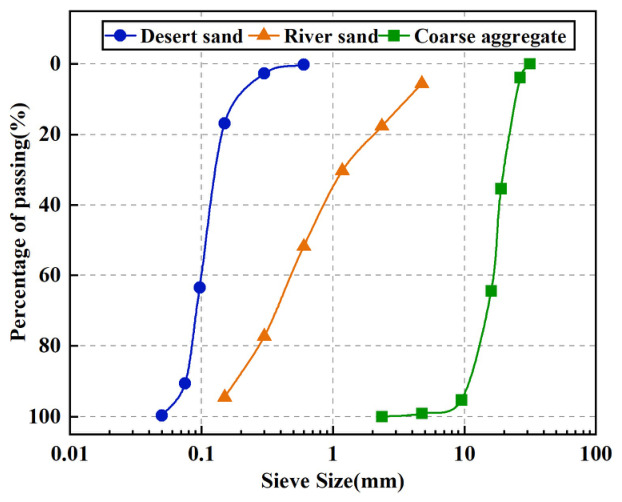
Aggregate grading curve (reproduced from [39]).

All raw materials used in this experiment are consistent with those in Ref. [39]; the cement is P.O 42.5 ordinary Portland cement produced by Tianshan Cement Plant in Urumqi, and its main physical performance indices are presented in Table 1. The fly ash employed was Class F, Grade I fly ash supplied by the Industry and Trade Branch of Xinjiang Tianfu Thermal Power Company. The fine aggregates consisted of washed medium sand from the Manas River in Xinjiang and desert sand from the Gurbantünggüt Desert; their morphological characteristics, main physical properties, and particle size distribution curves are shown in Figure 1, Table 2, and Figure 2, respectively. The coarse aggregate was continuously graded gravel, with its particle size distribution curve also given in Figure 2. A polycarboxylate superplasticizer with a water-reducing efficiency of 20% was used as the water reducer. The water-to-binder ratio was 0.4, and the mix proportion was determined on the basis of the previous research findings of our team [39]; the detailed mix proportions are listed in Table 3.

### 2.2. Specimen Design

**Figure 3:**
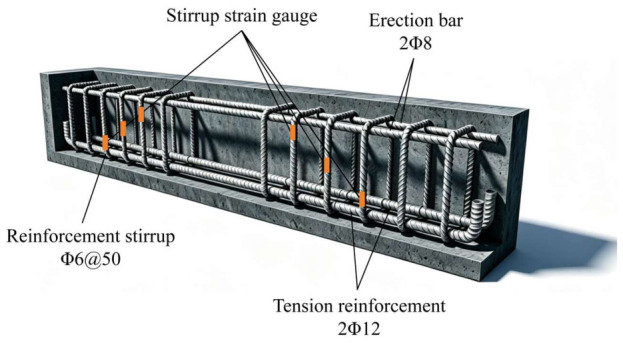
Detail of specimen.

Consistent with our previously published research [39], a total of 16 DSCBs were fabricated, all with uniform dimensions of 100 mm × 150 mm × 800 mm and 20 mm concrete cover was reserved The detailed construction drawings are shown in Figure 3, with all detailed design parameters summarized in Table 4. All reinforcements were HRB400 hot-rolled ribbed steel bars (manufacturer: Xinjiang Bayi Iron & Steel Co., Ltd., Urumqi, China). The tensile longitudinal bars were 2Φ12, the stirrups for supporting bars were 2Φ8, and the hoops were Φ6@50. The design value of the hoop tensile strength was fy = 360 MPa. The shear span–depth ratio was fixed at 1.7 for all specimens. To exclude the interference caused by longitudinal reinforcement ratio, stirrup ratio and shear span–depth ratio, all test specimens were designed with consistent cross-sectional sizes and reinforcement layouts, as well as a unified shear span–depth ratio. In this test, only two variables were set, namely the freeze–thaw cycle number (0, 25, 50, 75) and desert sand replacement proportion (0%, 20%, 40%, 60%). In this experiment, a two-factor coupled analysis framework was established by controlling the desert sand replacement ratio and the number of freeze–thaw cycles, so as to systematically reveal the influence mechanism of material and environmental factors on the shear performance of DSCBs. The specimen labeling convention is as follows: “DSCB-0-20” indicates a desert sand concrete beam subjected to 0 freeze–thaw cycles with a desert sand replacement ratio of 20%.

Standard 150 mm cubic specimens and 150 mm × 150 mm × 300 mm prism samples were prepared concurrently during the pouring of each test beam to determine fundamental concrete mechanical performances, including cubic compressive strength (*f*_cu_), splitting tensile strength (*f*_t_), axial compressive strength (*f*_c_), and elastic modulus (*E*_c_). These auxiliary concrete samples underwent identical curing regimes as the experimental beams [39]. All tested mechanical performance indicators are listed in Table 4, where each parameter corresponds to the averaged result obtained from three parallel replicate specimens.

### 2.3. Freeze–Thaw Cycling Test

Following the rapid freezing procedure outlined in the standard test code for long-term performance and durability of conventional concrete [40], accelerated freeze–thaw tests with water freezing and water thawing conditions were carried out via a TDRF-1 rapid freeze–thaw test apparatus (Tianjin Huida Experimental Instrument Co., Ltd., Tianjin, China). Throughout the testing process, the water level was kept 5 mm higher than the top surface of each specimen. A single freeze–thaw cycle lasted 2–4 h, with the thawing duration occupying no less than one-fourth of the total cycle time. The upper and lower temperature limits during the test were controlled at 7 °C ± 2 °C and −20 °C ± 2 °C, respectively. Upon the completion of 0, 25, 50, and 75 freeze–thaw cycles, the test samples were taken out and cleared of surface residues. After natural air drying, visual inspection and morphological damage observation were implemented for all specimens.

### 2.4. Loading System and Measurement Point Arrangement

**Figure 4:**
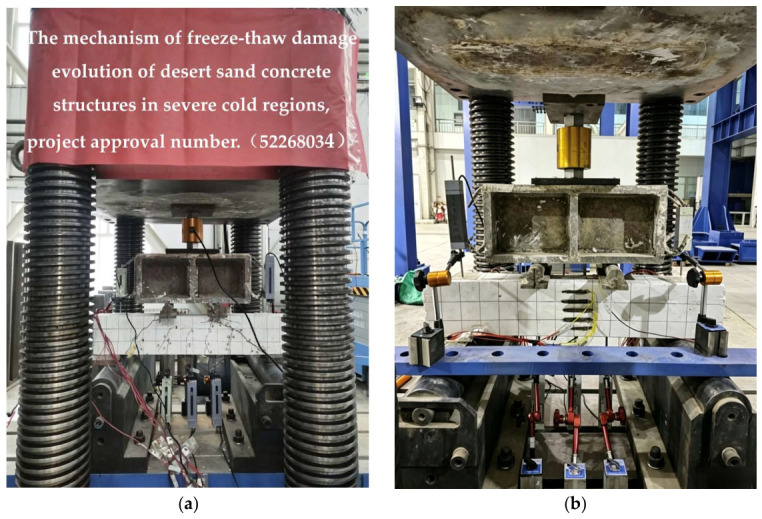
Test loading device and measuring point layout: (**a**) Front view of the loading device. (**b**) Rear view of the loading device.

A 5000 kN long-column compression testing machine (manufacturer: Changchun Testing Machine Co., Ltd., Changchun, China) was employed for four-point bending loading. Steel bearing plates (50 mm wide and 20 mm thick) were placed at the loading points and supports to prevent local crushing, and a fine sand leveling layer was applied on the concrete surface at the supports and loading locations. The loading setup is shown in Figure 4. To measure stirrup strains, three sets of stirrup strain gauges (type BMB120-3AA (Zhonghang Electronic Measuring Instruments (Xi’an) Co., Ltd., Xi’an, China)) were arranged along the line connecting the loading point to the support, as illustrated in Figure 3. For concrete strain measurement, five concrete strain gauges were uniformly distributed along the beam height at mid-span. To measure deflections, three displacement transducers were installed at the mid-span of the specimen, and two displacement transducers were placed at the supports; the arrangement is shown in Figure 4.

In accordance with the Standard for Test Methods of Concrete Structures [41], the specimens were loaded. The loading procedure adopted load control. During the preloading stage, a load of 5 kN was applied in three increments. In the formal loading stage, the load was first increased in increments of 10% of the ultimate bearing capacity; after the load reached 90% of the ultimate value, the increment was reduced to 5%. At each load-holding stage, the crack patterns and crack widths were recorded until specimen failure. Test data were automatically recorded using a TDS-530 static data acquisition system (Guangzhou Oumei Dadi Instrument Equipment Co., Ltd., Guangzhou, China), with continuous data collection throughout the testing process. The primary data collected included cracking load, ultimate load, mid-span deflection, stirrup strain, and concrete strain.

The determination of the diagonal-section cracking load followed the criteria below: (1) the load value is corresponding to the first visual observation of a diagonal crack within the shear span; and (2) in conjunction with strain data—when the stirrup strain in the shear span exhibited a sudden change or a marked increase in slope, this was also taken as an indication of diagonal cracking. The smaller value obtained from the two methods was adopted as the final cracking load. The final diagonal-section cracking load was taken as the load value at which the diagonal crack was first observed during loading, and the corresponding abrupt change point in the load–strain curve was simultaneously recorded for cross-verification.

## 3. Shear Behavior and Damage Mechanism of DSCB Under Freeze–Thaw Cycles

### 3.1. Failure Mode and Crack Distribution

**Figure 5:**
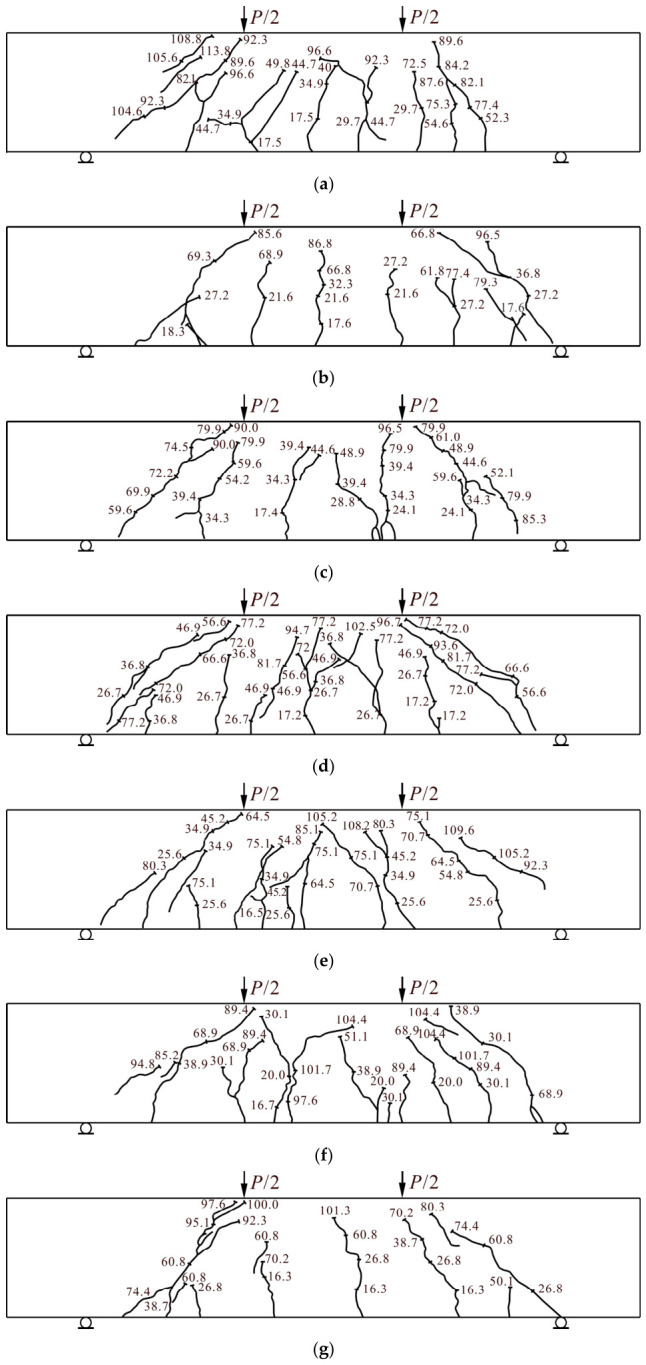

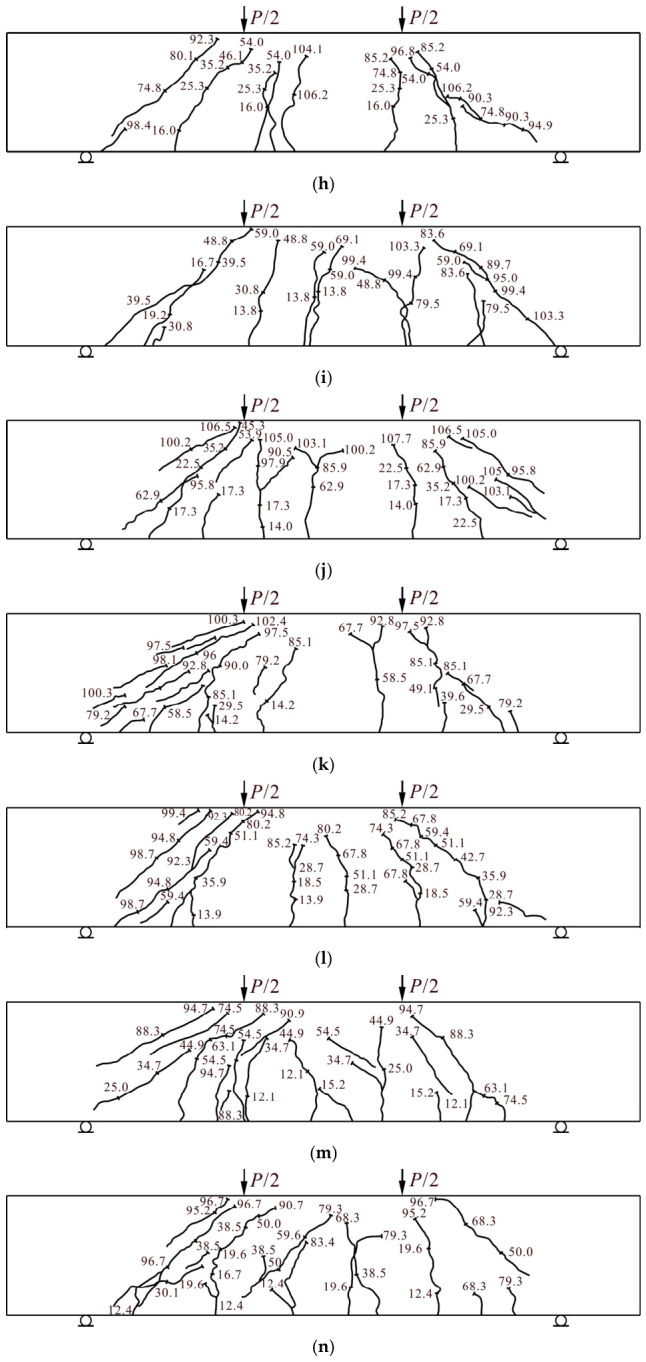

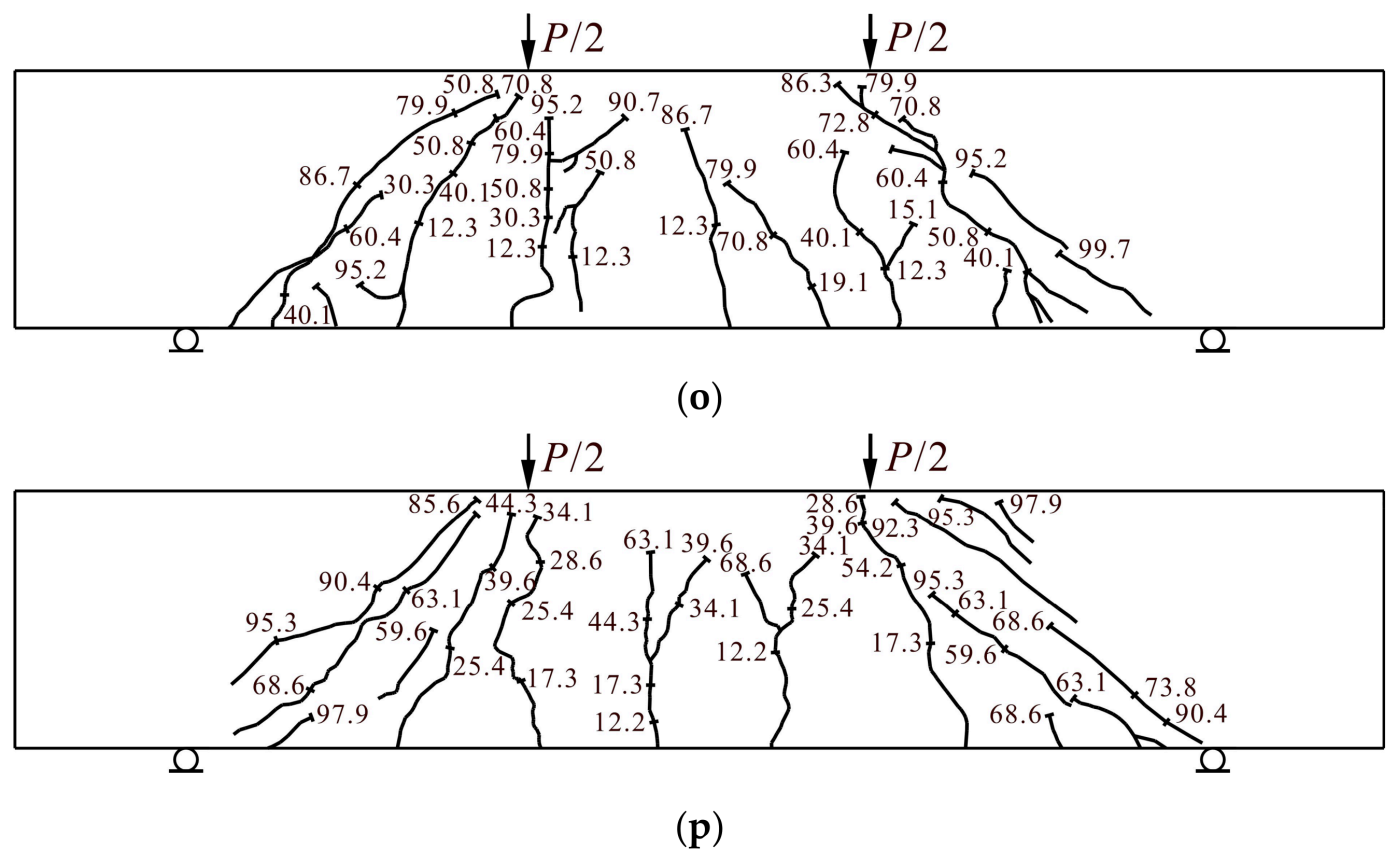
Crack development (Unit: kN): (**a**) DSCB-0-0; (**b**) DSCB-0-20; (**c**) DSCB-0-40; (**d**) DSCB-0-60; (**e**) DSCB-25-0; (**f**) DSCB-25-20; (**g**) DSCB-25-40; (**h**) DSCB-25-60; (**i**) DSCB-50-0; (**j**) DSCB-50-20; (**k**) DSCB-50-40; (**l**) DSCB-50-60; (**m**) DSCB-75-0; (**n**) DSCB-75-20; (**o**) DSCB-75-40; (**p**) DSCB-75-60 (reproduced from [39]).

Figure 5 shows the crack distribution and final failure modes of the specimens [39]. It can be seen from the figure that all specimens exhibited shear-compression failure, and the crack development pattern and failure mode of DSCBs are similar to those of ordinary concrete beams. The diagonal cracking load, shear capacity, and failure mode of the specimens are presented in Table 5. Regarding the detailed failure process of the specimens, When the load reached 11.3–14.9% of the ultimate load, a few vertical bending cracks first appeared at the mid-span of the beams, with lengths ranging from 28 to 68 mm and relatively small widths. When the load reached 17.9–33.2% of the ultimate load, fresh fractures kept generating within the pure bending zone, with their width and height expanding progressively. Several diagonal cracks emerged in the shear span and exhibited a slow growth trend. As the applied load rose to 34.4–65.5% of the ultimate bearing capacity, diagonal cracks in the shear span sprouted rapidly and propagated continuously, eventually evolving into the primary controlling cracks. When the load reached 76.1–91.4% of the ultimate load, diagonal cracks in the shear span quickly extended toward the loading points and supports, eventually forming continuous main diagonal cracks, resulting in the failure of the member.

The crack development process and failure severity varied among specimens due to the number of freeze–thaw cycles and the desert sand replacement ratio. As the number of freeze–thaw cycles increased, microcracks and pore defects inside the concrete accumulated continuously. The cracking load of the specimens gradually decreased, the initiation of diagonal cracks occurred earlier, and the propagation rate significantly increased, further highlighting the shear-compression behavior. Specimens subjected to 50 and 75 freeze–thaw cycles exhibited more diagonal cracks with noticeably larger widths [19].

Regarding the desert sand replacement ratio, specimens with low to medium replacement ratios could rely on stirrup confinement, longitudinal bar dowel action, and stress redistribution after crack penetration to effectively delay the development of principal diagonal cracks and maintain good deformability. In contrast, high replacement ratio specimens under freeze–thaw action experienced a significant reduction in shear span capacity and mechanical interlock between aggregates, making the principal diagonal cracks more likely to propagate rapidly and causing a marked decrease in member ductility [20].

Overall, although differences existed among specimen groups in terms of crack patterns, propagation rates, and damage severity, all specimens ultimately failed primarily through penetration of the principal diagonal cracks and local crushing of the concrete in the shear span, exhibiting a consistent shear-compression failure mode.

### 3.2. Diagonal-Section Cracking Behavior

(1) Effect of desert sand replacement ratio

For specimens without freeze–thaw action, the diagonal cracking load declines marginally as the desert sand replacement level rises. At a replacement rate of 60%, this index drops by roughly 3.0% compared with conventional concrete. The smooth surface and large specific surface area of desert sand particles are the main causes: they weaken the mechanical interlock among aggregates and introduce more initial microcracks inside the cement matrix.

After experiencing freeze–thaw cycles, the variation law of cracking load against desert sand content transforms into an upward trend followed by a downward trend. The maximum cracking load occurs at the replacement ratio of 20%, which surpasses the value of conventional concrete beams. To illustrate, after 75 freeze–thaw cycles, the cracking load of specimens with 20% desert sand replacement is 20.56 kN, while the counterpart for the group without desert sand is 19.83 kN. Once the replacement proportion goes beyond 40%, the cracking load drops sharply.

Moderate incorporation of desert sand (20%) exerts an excellent micro-filling effect. It can optimize the pore structure, compact the interfacial transition zone and cut down the quantity of freezable water. These positive effects mitigate freeze–thaw deterioration and enhance the anti-cracking performance of beam specimens. Nevertheless, when the replacement ratio is no less than 40%, the mixing water requirement of concrete increases obviously, accompanied by a raised effective water–cement ratio. In addition, fine desert sand particles will adhere to coarse aggregates and impair the interface bonding performance, ultimately leading to a substantial reduction in cracking load after freeze–thaw exposure [42,43].

(2) Effect of freeze–thaw cycles

For specimens with any given fixed desert sand replacement ratio, the diagonal-section cracking load decreases with increasing number of freeze–thaw cycles, although the magnitude of reduction varies with the replacement ratio. After 75 freeze–thaw cycles, the cracking loads of specimens with replacement ratios of 0%, 20%, 40%, and 60% decreased by 16.0%, 11.6%, 12.7%, and 14.2%, respectively. It should be noted that the slightly lower reduction in diagonal-section cracking load for the 60% replacement ratio specimens, compared with that of ordinary concrete, does not imply that they possess superior freeze–thaw resistance. This group of specimens already exhibited a lower cracking load prior to freeze–thaw exposure, suggesting that there may be more potential weak zones within them. Consequently, during subsequent freeze–thaw cycles, the damage development is more characterized by the propagation of existing defects rather than the formation of numerous new defects. Since their initial performance baseline is lower, the margin for further degradation is relatively limited, thus resulting in a somewhat smaller relative reduction. Nevertheless, after 75 freeze–thaw cycles, their cracking load remains lower than that of ordinary concrete specimens. When evaluating freeze–thaw performance, a comprehensive analysis should be carried out by combining the initial cracking load, the reduction magnitude, and the diagonal-section cracking load after freeze–thaw exposure. Among all replacement ratios, the 20% replacement ratio exhibits the smallest reduction, with the highest cracking loads both before and after freeze–thaw cycles, indicating the best freeze–thaw resistance. Mechanistically, freeze–thaw cycles cause the free water in concrete pores to repeatedly freeze and expand, generating freezing-induced tensile stresses. When these stresses exceed the local tensile strength of the matrix, microcracks are initiated. With increasing number of cycles, the cracks propagate and coalesce, leading to gradual deterioration of the tensile strength, elastic modulus, and bonding performance of the interfacial transition zone, which in turn causes diagonal cracks to appear earlier and the cracking load to decrease accordingly [13]. The 20% replacement ratio, owing to its densest internal structure and the lowest content of freezable water, is most effective in inhibiting the initiation and propagation of freeze–thaw cracks; hence, its cracking load decreases at the slowest rate [44].

### 3.3. Evolution Law of Shear Capacity

(1) Effect of desert sand replacement ratio

**Figure 6:**
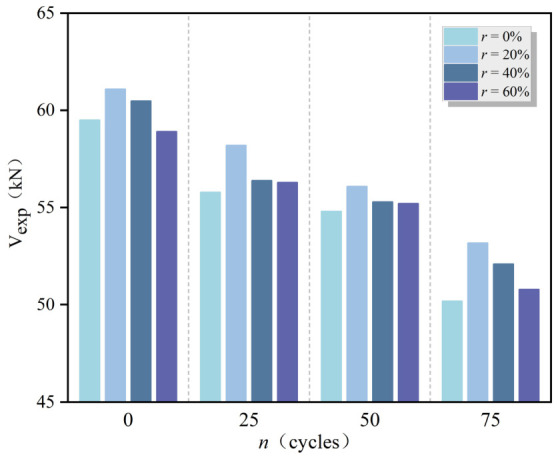
Effect of desert sand replacement ratio: *V*_exp_: Measured shear capacity (kN); *n*: Freeze–thaw cycles; *r*: Desert sand replacement ratio (%).

As presented in Figure 6, the shear capacity of all specimens exhibited a nonlinear variation with increasing *r*: it increased gradually when r was below 20% and decreased progressively when *r* exceeded 20%. The DSCBs with *r* = 20% exhibited the highest shear capacity under all freeze–thaw cycle conditions. This phenomenon was mainly attributed to the combined effects of the filling action of desert sand and the optimization of aggregate gradation. When *r* was 20%, an appropriate amount of fine desert sand particles effectively filled the voids between coarse aggregates and the cementitious matrix, significantly improving the compactness of the concrete matrix and reducing internal porosity, thereby enhancing the overall shear performance [42]. Compared with ordinary concrete beams, the shear capacity of DSCBs with *r* = 20% increased by 2.7%, 4.3%, 2.4%, and 6.0% following 0, 25, 50 and 75 freeze–thaw exposures. In addition, a reasonable replacement ratio of 20% optimized the aggregate gradation, making the internal structure denser along with bettering the interfacial bond properties, thereby further enhancing the shear capacity of the specimens. In contrast, when *r* exceeded 40%, excessive fine particles substantially increase the specific surface area of the aggregate. With a constant dosage of cementitious materials, more mixing water is required to ensure that the particle surfaces are adequately coated with paste, which consequently raises the effective water-to-binder ratio of the concrete. This increase tends to promote greater drying shrinkage and induce internal microcracks. At higher replacement ratios, the mechanical interlocking between aggregates is markedly weakened, and the frictional resistance and aggregate interlocking action on the crack plane are reduced, thereby compromising the shear performance. For example, under non-freeze–thaw conditions, the shear capacity of specimens with *r* = 60% was 1.0% lower than that of ordinary concrete beams. In addition, desert sand particles are relatively smooth, and a high replacement ratio further reduces the mechanical interlocking between aggregates, ultimately resulting in a continuous decline in shear capacity. Comprehensive analysis indicated that when *r* was 20%, the void-filling effect and interfacial structural integrity achieved the optimal balance, resulting in the best shear capacity [13].

(2) Effect of freeze–thaw cycles

**Figure 7:**
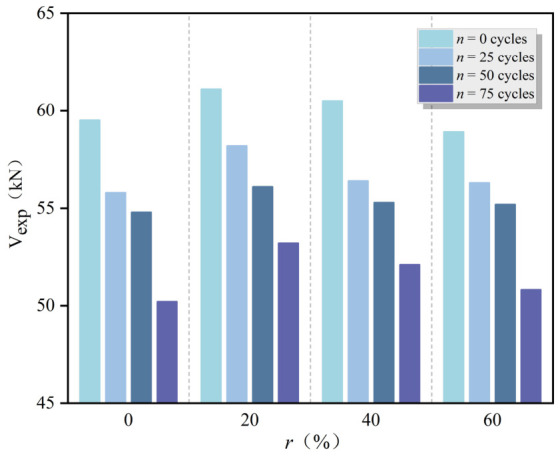
Effect of freeze–thaw cycles: *V*_exp_: Measured shear capacity (kN); *n*: Freeze–thaw cycles; *r*: Desert sand replacement ratio (%).

As presented in Figure 7, when *r* remained constant, the shear capacity of all specimens gradually decreased with increasing *n.* This was attributed to the continuous accumulation of internal damage and progressive stiffness degradation caused by freeze–thaw cycles. During a single freeze–thaw cycle, when pore water freezes and expands, tensile stress caused by frost heave forms inside the material. If the stress grew larger than the tensile strength at partial matrix areas, microcracks began to form. As *n* increased, the cracks continuously propagated, interconnected, and formed crack networks. The interfacial transition zone and aggregate interlocking effect were progressively weakened, ultimately leading to the gradual deterioration of shear capacity [44,45]. In terms of degradation magnitude, after 75 freeze–thaw cycles, the shear bearing capacities of specimens with replacement ratios *r* of 0%, 20%, 40%, and 60% decreased by 15.6%, 12.9%, 13.9%, and 13.8%, respectively, compared with their non-freeze–thaw counterparts. The overall degradation process exhibits pronounced nonlinear characteristics: during the initial stage of 0–25 cycles, the bearing capacity decreases relatively significantly; in the 25–50 cycle stage, the reduction rate of shear bearing capacity is comparatively slower than that in the subsequent 50–75 cycle stage, indicating that the damage evolution during this period is in a transitional state from initial damage accumulation to later-stage accelerated deterioration, rather than entering a phase of rapid unstable failure. It should be noted that this stage division is based on the variation patterns of macroscopic mechanical parameters such as shear bearing capacity and diagonal-section cracking load, rather than on direct observations of crack propagation behavior at the microscopic level.

It is noteworthy that the specimen with *r* = 20% exhibited the smallest reduction in shear capacity after 75 freeze–thaw cycles, at only 12.9%, indicating that DSCBs with a 20% replacement ratio possessed the best freeze–thaw resistance. The main reason is that a 20% replacement ratio produced a denser internal concrete structure, which effectively inhibited moisture ingress and alleviated frost-heaving pressure within pores. As a result, the initiation and propagation of freeze–thaw cracks were significantly delayed, enabling the specimens to better retain their shear capacity under freeze–thaw conditions [46].

### 3.4. Load–Stirrup Strain Response

(1) Effect of desert sand replacement ratio

**Figure 8:**
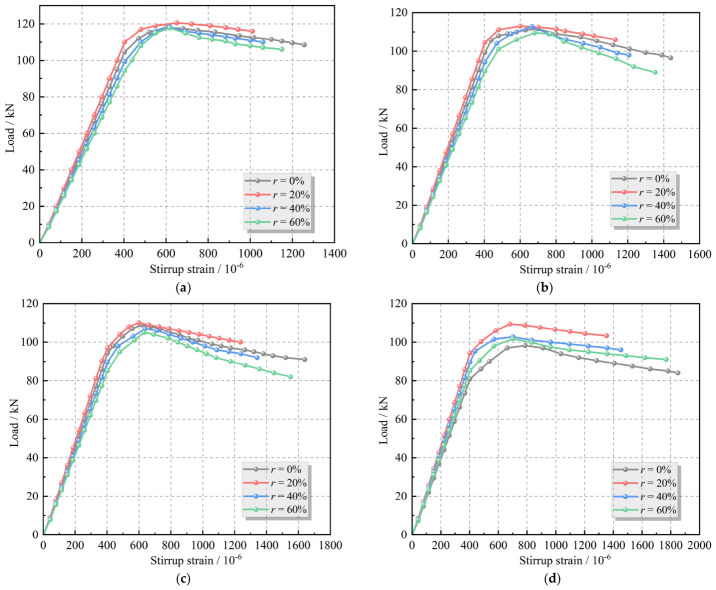
Load–stirrup strain curve for varying desert sand replacement ratio: (**a**) *n* = 0 cycles; (**b**) *n* = 25 cycles; (**c**) *n* = 50 cycles; (**d**) *n* = 75 cycles. *n*: Freeze–thaw cycles; *r*: Desert sand replacement ratio (%).

For the same number of freeze–thaw cycles *n*, the stirrup strain of DSCBs decreased with the incorporation of desert sand. The test results are presented in Figure 8. A comparative analysis was conducted based on the stirrup strains corresponding to the peak load of each specimen, and the reduction magnitudes are summarized in Table 6. As shown in Table 6, when the desert sand replacement ratio was 20%, the stirrup strain was relatively low with a comparatively larger reduction; the 40% replacement ratio exhibited an intermediate reduction, while the 60% replacement ratio showed the smallest reduction. It is evident that, under the test conditions of this study, the specimens with a 20% replacement ratio exhibited a lower stirrup strain level at similar loading stages. The reason is that desert sand particles are extremely fine. When incorporated at an appropriate dosage (approximately 20%), they can exert a micro-filler effect, uniformly filling capillary pores in the cement matrix and voids between natural sand particles, optimizing particle gradation, reducing total porosity, refining pore structure, and particularly decreasing harmful large pores. Meanwhile, the fine particles can improve the compactness of the aggregate-cement paste interfacial transition zone (ITZ), promote secondary hydration reactions to generate C-S-H gel, and enhance the overall stiffness and freeze–thaw resistance of the matrix [19]. This enables the concrete itself to bear more stress under the same external load, reducing the stress carried by the stirrups and significantly decreasing their strain. When the replacement ratio increased to 40%, the filling effect still existed, but the water demand began to increase. Some negative effects offset the advantages, resulting in a smaller reduction. When the replacement ratio reached 60%, the excessively large specific surface area of desert sand significantly increased the water demand, leading to a higher effective water–cement ratio, which in turn increased porosity and weakened interfacial bonding. In addition, excessively fine particles coated the coarse aggregates and hindered the effective anchorage of cement paste, increasing the initial defects in concrete and reducing its freeze–thaw resistance. Therefore, the reduction in stirrup strain was very limited and even approached or exceeded that of specimens with a 0% replacement ratio [47,48].

(2) Effect of freeze–thaw cycles

**Figure 9:**
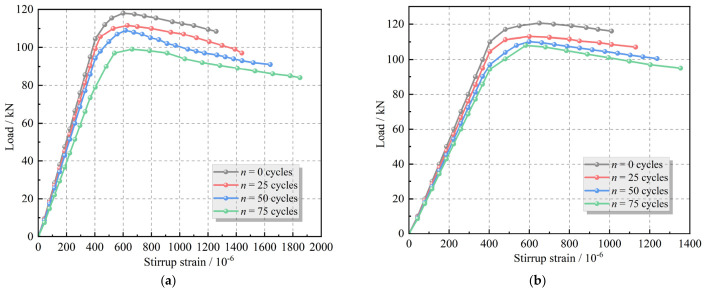

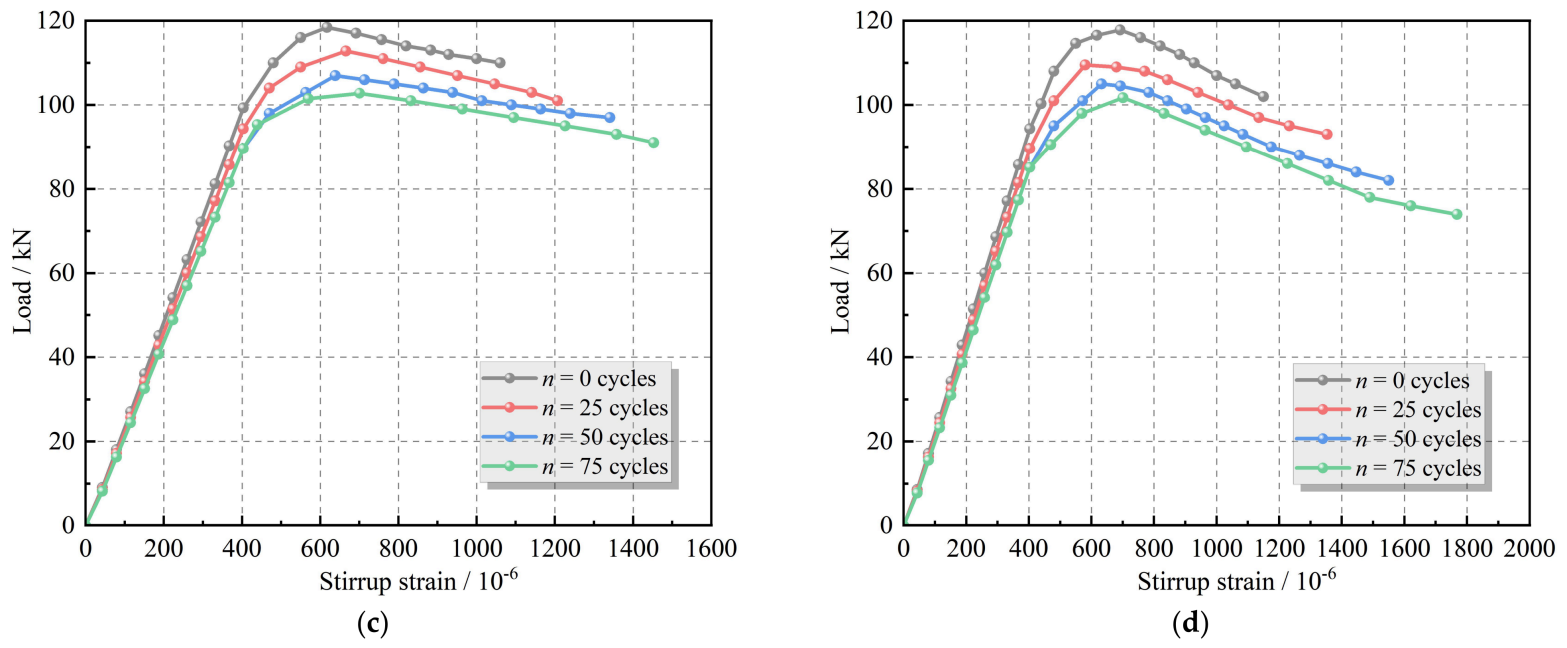
Load–stirrup strain curve for varying freeze–thaw cycles: (**a**) *r* = 0%; (**b**) *r* = 20%; (**c**) *r* = 40%; (**d**) *r* = 60%. *n*: Freeze–thaw cycles; *r*: Desert sand replacement ratio (%).

For the same replacement ratio the stirrup strain of DSCB exhibited an increasing trend with the number of freeze–thaw cycles, although the rates of increase varied. The test results are presented in Figure 9. A comparative analysis was conducted based on the stirrup strains corresponding to the peak load of each specimen, and the specific magnitudes of increase are given in Table 7. As shown in Table 7, within the test range of this study, the growth rates of stirrup strain differed among specimens with various replacement ratios. Specifically, the specimens with a 20% replacement ratio showed a relatively slower increasing trend, while those with a 60% replacement ratio exhibited a faster increase. The internal pore water inside concrete freezes and expands repeatedly and then thaws and shrinks during cyclic frost action, producing recurring crystallization pressure. Once the pressure surpasses the tensile strength of concrete matrix, tiny cracks start to appear. With ongoing cycles, these fractures expand and connect with one another, leading to obvious declines in concrete elastic modulus and interfacial bonding strength. Consequently, a greater proportion of the load originally carried by the concrete was transferred to the stirrups, leading to higher stirrup strains [49]. After incorporating 20% desert sand, the pore structure was refined, pore connectivity was reduced, and the amount of freezable water decreased. As a result, the freezing pressure was more easily absorbed by the elastic deformation of pore walls, inhibiting the propagation of microcracks. Meanwhile, secondary hydration reactions could partially heal early-stage cracks, resulting in the slowest damage accumulation rate and the smallest increase in stirrup strain [50]. In contrast, specimens with a 60% replacement ratio had a higher initial porosity and weaker interfaces, resulting in greater damage during each freeze–thaw cycle and exhibiting accelerated deterioration characteristics. In the later stage of freeze–thaw cycling, the concrete may have experienced significant spalling, causing the stirrups to bear nearly all the load and resulting in the largest increase in strain. Ordinary concrete with 0% replacement lacked the filling effect of fine particles and exhibited a moderate damage rate. Although the 40% replacement specimens still benefited from some filling effect, the adverse effects caused by excessive fine particles had begun to appear; therefore, the increase in strain was between those of the 0% and 20% replacement specimens [51].

## 4. Prediction Model for Shear Capacity of DSCB Under Freeze–Thaw Environment

### 4.1. Calculation Model and Modification for Diagonal-Section Cracking Load

Since no dedicated design code for DSC structures has been established within the current engineering design system in China, there is currently no specific calculation method available for evaluating the mechanical performance of DSCBs. Therefore, the effects of freeze–thaw cycling on the diagonal crack development of such beams cannot be quantitatively evaluated with high precision. For this reason, the calculation expressions adopted by the current concrete structure design specification [38] were adopted in this study to preliminarily calculate the diagonal cracking load of DSCBs after freeze–thaw exposure as follows:

(1) $$V_{\mathrm{cr}}=1.8bh_{0}f_{t}/(\lambda +1.3)$$

where *V*_cr_ is the diagonal-section cracking load (kN); *h*_0_ is the effective depth of the section (mm); *f*_t_ is the tensile strength of concrete (MPa); and *λ* is the shear span-to-depth ratio (dimensionless).

The definitions of all parameters in the equation are specified in the national concrete structural design specification [38]. The calculated diagonal cracking load, *V*_cr,cal_, and the experimentally measured value, *V*_cr,exp_, are presented in Table 8.

As presented in Table 8, average coefficient between numerically calculated cracking loads and measured results, *V*_cr,cal_/*V*_cr,exp_, was 0.889, with a standard deviation of 0.022 and a coefficient of variation of 0.025. It is feasible to calculate the diagonal cracking load using Equation (1); however, the results are overly conservative and cannot accurately reflect the actual diagonal cracking behavior of members subjected to the coupled impacts induced by freeze–thaw deterioration and desert sand substitution. Therefore, the diagonal cracking load equation provided in the current code [38] is not applicable to DSCBs after freeze–thaw exposure.

To establish a prediction model for the diagonal-section cracking load under the combined effects of freeze–thaw cycles and desert sand replacement ratio, a multiple linear least squares regression analysis was conducted on the test results. In the regression process, the residual sum of squares between the modified calculated values and the experimentally measured values was adopted as the objective function for parameter optimization. The objective function is expressed as follows:

(2) $$Q=\sum_{i=1}^{16}(V_{\mathrm{cr},\mathrm{cor},i}-V_{\mathrm{cr},\exp,i}{)}^{2}$$

where *Q* is the residual sum of squares; *V*_cr,exp,i_ is the experimental value of the”i”-th specimen (kN); *V*_cr,cor,i_ is the fitted calculated value of the”i”-th specimen (kN).

A two-parameter correction coefficient *ξ*(*r*,*n*) is introduced to modify the code-specified calculation formula. The modified calculated values of the cracking load, *V*_cr,cor_, are presented in Table 8. Here, the desert sand replacement ratio *r* is expressed as a decimal in the calculation, taking values of 0, 0.2, 0.4, and 0.6 (corresponding to actual replacement ratios of 0%, 20%, 40%, and 60%, respectively); the number of freeze–thaw cycles *n* is given in cycles, with values of 0, 25, 50, and 75.

Through regression analysis of the diagonal-section cracking load data, the expression for the correction coefficient *ξ*(*r*,*n*) as a function of the desert sand replacement ratio *r* and the number of freeze–thaw cycles *n* is obtained as follows:

(3) ξ(r,n)=1.164+0.091r−0.00107n

where *ξ*(*r*,*n*) is dimensionless; *r* is the desert sand replacement ratio, taking values of 0, 0.2, 0.4, and 0.6; *n* is the number of freeze–thaw cycles, taking values of 0, 25, 50, and 75. The constant term 1.164, the coefficient of desert sand replacement ratio 0.091, and the coefficient of freeze–thaw cycles −0.00107 are all dimensionless quantities.

In summary, the calculation formula for the diagonal-section cracking load of DSCB after freeze–thaw cycles is given as follows:

(4) $$V_{\mathrm{cr}}=1.8\xi bh_{0}f_{t}/(\lambda +1.3)$$

As shown in Table 8, the mean value of the ratio of the modified calculated value to the experimental value of the diagonal-section cracking load, *V*_cr,cor_/*V*_cr,exp_ is 1.024, with a standard deviation of 0.022, a coefficient of variation of 0.021, and a coefficient of determination R^2^ of 0.926. The statistical parameters exhibit relatively small fluctuations, which fully demonstrates that the calculated values from the modified formula are in good agreement with the experimental measurements, and that the modified model is of high reliability, indicating favorable fitting stability within the parameter range of this study.

It should be noted that the prediction model proposed in this paper is established based on the test results of 16 reinforced desert sand concrete beams and is therefore an empirical regression model. Owing to the limited number of specimens—with only one specimen per testing condition—residual analysis, parameter significance testing, and confidence interval analysis were not conducted in this study, nor was independent external validation data available. Consequently, the statistical uncertainty of the model remains subject to certain limitations. The proposed model is applicable to reinforced desert sand concrete beams with conditions similar to those of the present study, including comparable cross-sectional dimensions, reinforcement detailing, shear span-to-depth ratio, concrete strength grade, desert sand replacement ratio (0, 0.2, 0.4, and 0.6), and number of freeze–thaw cycles (0, 25, 50, and 75). For members beyond the aforementioned scope, further experimental verification is required to confirm the applicability of the model.

### 4.2. Calculation Model and Modification for Shear Capacity

At present, no design code has been established in China specifically for DSC structures. Therefore, the corresponding equations provided in the current Code for Design of Concrete Structures [38] were adopted in this study to preliminarily calculate the shear capacity of DSCBs after freeze–thaw exposure as follows:

(5) $$V=V_{c}+V_{\mathrm{sv}}=\frac{1.75}{1+\lambda }f_{t}bh_{0}+f_{\mathrm{yv}}\frac{A_{\mathrm{sv}}}{S}h_{0}$$

In the equation, *V* is the design shear capacity, kN; *V*_c_ is the shear capacity provided by concrete, kN; *V*_sv_ is the shear capacity provided by stirrups, kN; *h*_0_ is the effective depth of the beam section, mm; *f*_yv_ is the design tensile strength of stirrups, Mpa; *A*_sv_ is the cross-sectional area of stirrups, mm^2^; and *S* is the stirrup spacing, mm. The measured mechanical properties of the materials were adopted in the calculations (see Table 4).

The experimental values and code-calculated values are presented in Table 9, where *V*_exp_ is the experimental shear capacity and *V*_cal_ is the value calculated using the code equation.

As presented in Table 9, the mean value of *V*_cal_/*V*_exp_ was 0.818, the standard deviation is 0.036, and the variation coefficient reaches 0.044. This indicates that the values calculated according to the current code [38] show poor agreement with the experimental results and are not suitable for DSCBs subjected to cyclic freezing and thawing. For the current investigation, the concrete specimens were prepared by replacing natural river sand with desert sand and were subjected to varying freeze–thaw exposure levels. The influence of these factors was mainly reflected in the tensile strength of concrete, *f*_t_. Therefore, it is necessary to modify *V*c in Equation (5) accordingly, so that the equation is more suitable for predicting the shear capacity of desert sand concrete beams after freeze–thaw exposure. Based on the above analysis, a modification coefficient *η*(*r,n*), related to the desert sand replacement ratio *r* and the number of freeze–thaw cycles *n*, was introduced to modify the concrete shear contribution *V*_c_ in the current code [38], where the desert sand replacement ratio *r* is expressed as a decimal in the calculation, taking values of 0, 0.2, 0.4, and 0.6 (corresponding to actual replacement ratios of 0%, 20%, 40%, and 60%, respectively); the number of freeze–thaw cycles *n* is given in cycles, with values of 0, 25, 50, and 75.

In this study, the multiple linear least squares method was employed to perform regression analysis on the test results. During the regression process, the residual sum of squares between the modified calculated values and the experimentally measured values was adopted as the objective function for parameter optimization. The objective function is expressed as follows:

(6) $$Q=\sum_{i=1}^{16}(V_{\mathrm{cor},i}-V_{\exp,i}{)}^{2}$$

where *Q* is the residual sum of squares; *V*_cor,i_ is the experimental value of the”i”-th specimen (kN); *V*_exp,i_ is the fitted calculated value of the”i”-th specimen (kN).

Through regression analysis of the shear bearing capacity values in Table 9, the expression for the correction coefficient *η*(*r,n*) as a function of the desert sand replacement ratio *r* and the number of freeze–thaw cycles *n* is obtained as follows:

(7) η(r,n)=1.7+0.17r−0.004n

where *η*(*r,n*) is dimensionless; *r* is the desert sand replacement ratio, taking values of 0, 0.2, 0.4, and 0.6; *n* is the number of freeze–thaw cycles, taking values of 0, 25, 50, and 75. The constant term 1.7, the coefficient of desert sand replacement ratio 0.17, and the coefficient of freeze–thaw cycles −0.004 are all dimensionless quantities.

Accordingly, the shear capacity prediction formula for DSCBs after freeze–thaw exposure is given as follows:

(8) $$V_{\mathrm{cor}}=\eta (r,n)\frac{1.75}{1+\lambda }f_{t}bh_{0}+f_{\mathrm{yv}}\frac{A_{\mathrm{sv}}}{S}h_{0}$$

As shown in Table 6, the mean value of the ratio of the modified calculated value to the experimental value, *V*_cor_/*V*_exp_ is 1.023, with a standard deviation of 0.018, a coefficient of variation of 0.017, and a coefficient of determination R^2^ of 0.907. This indicates that the modified formula can predict the shear bearing capacity of desert sand concrete beams after freeze–thaw cycles with reasonable accuracy, showing good agreement with the experimental results.

It should be noted that the prediction models for the diagonal-section cracking load and the shear bearing capacity proposed in this paper are empirical models established based on the test data of this study. Since both model parameter fitting and model evaluation were conducted using the same set of experimental data, the high consistency between the calculated and experimental values primarily reflects the fitting capability of the model to the existing test data, rather than fully representing its predictive capability for independent data. The applicability of the models is constrained by the test parameter conditions. These models are applicable to desert sand concrete beams with structural characteristics similar to those of the present study, including comparable cross-sectional dimensions, longitudinal reinforcement ratio, stirrup configuration, shear span-to-depth ratio, concrete strength grade, desert sand replacement ratio (0–60%), and number of freeze–thaw cycles (0–75). For members beyond the aforementioned parameter ranges, the applicability remains to be further verified by additional research.

## 5. Conclusions

This study explored the shear performance of DSCBs subjected to freeze–thaw conditions, and the main findings are summarized as follows:

(1) All DSCBs failed in a shear-compression mode, and their crack propagation characteristics and failure patterns were generally similar to those of ordinary concrete beams. However, as *n* increased, diagonal cracks appeared earlier, propagated more rapidly, and exhibited significantly greater numbers and widths.
(2) When the desert sand replacement ratio (*r*) was 20%, DSCBs exhibited the best overall mechanical performance under all freeze–thaw conditions, with a maximum increase of 6.0% in shear capacity and a maximum reduction of 26.8% in stirrup strain.
(3) Freeze–thaw cycling significantly deteriorated the shear performance of desert sand concrete beams. With increasing freeze–thaw cycles, the shear capacity continuously decreased and the deformation capacity gradually weakened. Additional frost-resistant design measures are therefore required under severe freeze–thaw conditions. After 75 freeze–thaw cycles, the shear capacities of specimens with desert sand replacement ratios of 0%, 20%, 40%, and 60% decreased by 15.6%, 12.9%, 13.9%, and 13.8%, respectively, while the corresponding stirrup strains increased by 47.2%, 34.1%, 37.1%, and 53.7%.
(4) By introducing correction coefficients related to the desert sand replacement ratio *r* and the number of freeze–thaw cycles *n*, calculation formulas for the diagonal-section cracking load and the shear bearing capacity of DSCBs were established based on the experimental data. The calculated values from these formulas are in good agreement with the experimental measurements, indicating that the proposed formulas can be used for preliminary estimation of the shear performance of desert sand concrete beams within the parameter range of this study. However, they do not yet possess general applicability for direct use in engineering design. Further verification and refinement are still required in future work by incorporating more experimental data and various structural conditions.
(5) The present study primarily investigated the effects of desert sand replacement ratio and the number of freeze–thaw cycles on the shear performance of reinforced concrete beams, while other parameters such as cross-sectional dimensions, longitudinal reinforcement ratio, stirrup ratio, and shear span-to-depth ratio were kept constant. Therefore, the conclusions and prediction models obtained in this study are mainly applicable to members within the parameter range investigated. Future research should further consider factors such as size effect, different reinforcement ratios, various shear span-to-depth ratios, and higher levels of freeze–thaw damage, in order to establish shear performance prediction methods with broader applicability.

## Figures and Tables

**Table 1:** Main physical properties of cement.

Cement	Standard Consistency Water Consumption/%	Soundness	Setting Time/h		Compressive Strength/Mpa		Flexural Strength/Mpa	
			Initial Setting Time	Final Setting Time	3 d	28 d	3 d	28 d
P.O 42.5	28.8	Qualified	2.7	4	25.5	49.0	5.0	7.4

**Table 2:** Main physical properties of fine aggregate (reproduced from [39]).

Category	Apparent Density (kg/m^3^)	Bulk Density (kg/m^3^)	Porosity (%)	Clay Content (%)	Water Ratio (%)
River sand	2038	1350	45	2.2	1.9
Desert sand	2630	1615	35	1.9	1.5

**Table 3:** Mix proportion of desert sand concrete (reproduced from [39]).

Desert Sand Replacement Rate *r*/%	Material Consumption/(kg·m^−3^)						
	Water	Cement	Fly Ash	Superplasticizer	Coarse Aggregate	River Sand	Desert Sand
0	160	370	30	1.6	1288	552.0	0
20	160	370	30	1.6	1288	441.6	110.4
40	160	370	30	1.6	1288	331.2	220.8
60	160	370	30	1.6	1288	220.8	331.2

**Table 4:** Design parameters of the specimens (reproduced from [39]).

Specimen	Cross-Section *b* × *h* × *l*/mm	*λ*	*ρ*/%	Stirrups	*r*/%	*n/*Times	*f*_cu_/MPa	*f*_t_/MPa	*f*_c_/MPa	*E*_c_/GPa
DSCB-0-0	100 × 150 × 800	1.7	1.51%	Φ6@50	0	0	44.5	2.85	21.81	38.7
DSCB-0-20	100 × 150 × 800	1.7	1.51%	Φ6@50	20	0	42.7	2.75	20.92	37.3
DSCB-0-40	100 × 150 × 800	1.7	1.51%	Φ6@50	40	0	41.6	2.68	20.38	36.5
DSCB-0-60	100 × 150 × 800	1.7	1.51%	Φ6@50	60	0	40.3	2.60	19.75	35.8
DSCB-25-0	100 × 150 × 800	1.7	1.51%	Φ6@50	0	25	37.6	2.53	18.42	34.4
DSCB-25-20	100 × 150 × 800	1.7	1.51%	Φ6@50	20	25	38.5	2.68	18.87	34.2
DSCB-25-40	100 × 150 × 800	1.7	1.51%	Φ6@50	40	25	38.1	2.62	18.67	33.8
DSCB-25-60	100 × 150 × 800	1.7	1.51%	Φ6@50	60	25	37.9	2.58	18.57	33.5
DSCB-50-0	100 × 150 × 800	1.7	1.51%	Φ6@50	0	50	37.3	2.51	18.28	32.9
DSCB-50-20	100 × 150 × 800	1.7	1.51%	Φ6@50	20	50	37.5	2.60	18.38	32.7
DSCB-50-40	100 × 150 × 800	1.7	1.51%	Φ6@50	40	50	37.4	2.55	18.33	32.3
DSCB-50-60	100 × 150 × 800	1.7	1.51%	Φ6@50	60	50	37.1	2.53	18.18	31.8
DSCB-75-0	100 × 150 × 800	1.7	1.51%	Φ6@50	0	75	33.1	2.41	16.22	31.7
DSCB-75-20	100 × 150 × 800	1.7	1.51%	Φ6@50	20	75	34.7	2.53	17.00	31.5
DSCB-75-40	100 × 150 × 800	1.7	1.51%	Φ6@50	40	75	34.2	2.50	16.76	31.3
DSCB-75-60	100 × 150 × 800	1.7	1.51%	Φ6@50	60	75	33.5	2.45	16.42	31.1

Note: “*λ*” is the shear span ratio; “*ρ*” is the reinforcement ratio; “*r*” is the desert sand replacement ratio; “*n*” is the number of freeze–thaw cycles; “*f*_cu_” is the cube compressive strength of concrete, “*f*_t_” is the tensile strength of concrete, “*f*_c_” is the axial compressive strength of concrete, and “*E*_c_” is the elastic modulus of concrete; the same applies below.

**Table 5:** Shear performance parameters and failure modes of test beams.

Specimen	Diagonal Cracking Load *V*_cr,exp_/kN	Shear Bearing Capacity *V*_exp_/kN	Failure Mode
DSCB-0-0	23.61	59.50	shear compression
DSCB-0-20	23.25	61.10	shear compression
DSCB-0-40	23.12	60.50	shear compression
DSCB-0-60	22.91	58.90	shear compression
DSCB-25-0	21.53	55.80	shear compression
DSCB-25-20	22.65	58.20	shear compression
DSCB-25-40	22.23	56.40	shear compression
DSCB-25-60	21.96	56.30	shear compression
DSCB-50-0	20.87	54.80	shear compression
DSCB-50-20	21.56	56.10	shear compression
DSCB-50-40	21.33	55.30	shear compression
DSCB-50-60	20.95	55.20	shear compression
DSCB-75-0	19.83	50.20	shear compression
DSCB-75-20	20.56	53.20	shear compression
DSCB-75-40	20.18	52.10	shear compression
DSCB-75-60	19.65	50.80	shear compression

**Table 6:** Variation magnitude of stirrup strain of DSCB relative to ordinary concrete beams under different desert sand replacement ratios.

	*r*/%	20	40	60
*n*/Cycles				
0		−19.6%	−15.7%	−8.5%
25		−21.5%	−16.1%	−6.0%
50		−24.6%	−18.2%	−5.5%
75		−26.8%	−21.5%	−4.4%

Note: Negative values indicate a decrease compared to ordinary concrete beams.

**Table 7:** Variation magnitude of stirrup strain of DSCB relative to ordinary concrete beams under different freeze–thaw cycles.

	*n*/Cycles	25	50	75
*r*/%				
0		14.5%	30.5%	47.2%
20		11.9%	22.5%	34.1%
40		13.9%	26.5%	37.1%
60		17.7%	34.8%	53.7%

Note: The values in the figure indicate an increase compared to ordinary concrete beams.

**Table 8:** Comparison between code-calculated diagonal cracking load and experimental values.

Specimen	*V*_cr,exp_/kN	*V*_cr,cal_/kN	*V*_cr,cal_/*V*_cr,exp_	*V*_cr,cor_/kN	*V*_cr,cor_/*V*_cr,exp_
DSCB-0-0	23.61	21.20	0.898	24.68	1.045
DSCB-0-20	23.25	20.46	0.880	24.19	1.040
DSCB-0-40	23.12	19.95	0.863	23.94	1.036
DSCB-0-60	22.91	19.34	0.844	23.57	1.029
DSCB-25-0	21.53	18.82	0.874	21.41	0.994
DSCB-25-20	22.65	19.94	0.880	23.04	1.017
DSCB-25-40	22.23	19.49	0.877	22.88	1.029
DSCB-25-60	21.96	19.20	0.874	22.88	1.042
DSCB-50-0	20.87	18.67	0.895	20.74	0.994
DSCB-50-20	21.56	19.34	0.897	21.83	1.013
DSCB-50-40	21.33	18.97	0.889	21.76	1.020
DSCB-50-60	20.95	18.82	0.898	21.93	1.047
DSCB-75-0	19.83	17.93	0.904	19.43	0.980
DSCB-75-20	20.56	18.82	0.916	20.74	1.009
DSCB-75-40	20.18	18.60	0.922	20.83	1.032
DSCB-75-60	19.65	18.23	0.928	20.75	1.056
Average			0.889		1.024
Standard deviation			0.022		0.022
Coefficient of variation			0.025		0.021

**Table 9:** Comparison between code-calculated shear capacity and experimental values.

Specimen	*V*_exp_/kN	*V*_cal_/kN	*V*_cal_/*V*_exp_	*V*_cor_/kN	*V*_cor_/*V*_exp_
DSCB-0-0	59.50	47.32	0.795	62.77	1.055
DSCB-0-20	61.10	46.56	0.772	62.21	1.018
DSCB-0-40	60.50	45.98	0.777	61.91	1.023
DSCB-0-60	58.90	45.36	0.770	61.49	1.044
DSCB-25-0	55.80	45.51	0.816	57.67	1.034
DSCB-25-20	58.20	45.47	0.805	58.29	1.002
DSCB-25-40	56.40	45.08	0.799	58.33	1.034
DSCB-25-60	56.30	44.74	0.795	58.43	1.038
DSCB-50-0	54.80	45.43	0.829	55.52	1.013
DSCB-50-20	56.10	45.20	0.821	55.86	0.996
DSCB-50-40	55.30	44.89	0.816	56.05	1.014
DSCB-50-60	55.20	44.53	0.812	56.14	1.017
DSCB-75-0	50.20	44.24	0.901	51.84	1.033
DSCB-75-20	53.20	44.43	0.859	52.76	0.992
DSCB-75-40	52.10	44.02	0.856	52.81	1.014
DSCB-75-60	50.80	43.56	0.857	52.75	1.038
Average			0.818		1.023
Standard deviation			0.036		0.018
Coefficient of variation			0.044		0.017

## Data Availability

The original contributions presented in this study are included in the article. Further inquiries can be directed to the corresponding authors.
